# Medial Inguino-Femoral Lymphadenectomy for Vulvar Cancer: An Approach to Decrease Lymphedema without Compromising Survival

**DOI:** 10.3390/cancers13225806

**Published:** 2021-11-19

**Authors:** Neville F. Hacker, Ellen Barlow, Stephen Morrell, Katrina Tang

**Affiliations:** 1 Gynaecological Cancer Centre, Royal Hospital for Women, Sydney 2031, Australia; ellen.barlow@health.nsw.gov.au; 2 School of Women’s and Children’s Health, University of New South Wales, Sydney 2033, Australia; 3 School of Population Health, University of New South Wales, Sydney 2033, Australia; stephen.morrell@unsw.edu.au; 4 Department of Anatomical Pathology, Prince of Wales Hospital, Sydney 2031, Australia; katrina.tang@health.nsw.gov.au

**Keywords:** inguino-femoral lymphadenectomy, lymphedema, vulvar cancer, medial inguinal nodes, lateral inguinal nodes

## Abstract

**Simple Summary:**

This prospective study has demonstrated that if the inguinal nodes medial to the lateral border of the femoral artery and the femoral nodes are negative in patients with vulvar cancer, the chances of having a positive node lateral to the artery are less than 1 in 10,000. About one-third of groin nodes are situated lateral to the femoral artery, so leaving these nodes in situ if the medial nodes are negative should significantly decrease the incidence and severity of lower limb lymphedema, without compromising survival.

**Abstract:**

Background: Lower limb lymphedema is a long-term complication of inguino-femoral lymphadenectomy and is related to the number of lymph nodes removed. Our hypothesis was that lymph nodes lateral to the femoral artery could be left in situ if the medial nodes were negative, thereby decreasing this risk. Methods: We included patients with vulvar cancer of any histological type, even if the cancer extended medially to involve the urethra, anus, or vagina. We excluded patients whose tumor extended (i) laterally onto the thigh, (ii) posteriorly onto the buttocks, or (iii) anteriorly onto the mons pubis. After resection, the inguinal nodes were divided into a medial and a lateral group, based on the lateral border of the femoral artery. Results: Between December 2010 and July 2018, 76 patients underwent some form of groin node dissection, and data were obtained from 112 groins. Approximately one-third of nodes were located lateral to the femoral artery. Positive groin nodes were found in 29 patients (38.2%). All patients with positive nodes had positive nodes medial to the femoral artery. Five patients (6.6%) had positive lateral inguinal nodes. The probability of having a positive lateral node given a negative medial node was estimated to be 0.00002. Conclusion: Provided the medial nodes are negative, medial inguino-femoral lymphadenectomy may suffice and should reduce lower limb lymphedema without compromising survival.

## 1. Introduction

The status of the groin lymph nodes is the single most important prognostic factor for patients with vulvar cancer [1]. The only way to be certain of the status of these nodes is to perform a complete inguino-femoral lymphadenectomy, but an inevitable consequence of this operation for many patients is lower limb lymphedema. Because it is a lifelong affliction, several attempts have been made to reduce the incidence of lymphedema over almost 50 years.

The first attempts in the mid-1970s involved trying to define a “microinvasive vulvar cancer” for which lymph node dissection could be completely omitted [2,3]. A “microinvasive” vulvar cancer was defined as one which had a maximum diameter of 20 mm and a maximum depth of invasion of 5 mm. Over the next decade, it became apparent that the only patients for whom there was virtually no risk of lymph node metastases were those whose cancers were 20 mm or less in diameter, with invasion no greater than 1 mm [4]. Such patients were subsequently classified by FIGO as having stage IA disease in 1994.

The second attempt was to perform a modified groin dissection. In 1979, DiSaia et al. reported “superficial inguinal node dissection”, removing only nodes above the cribriform fascia, for squamous cell carcinomas 10 mm or less in diameter with stromal invasion of 5 mm or less [5]. It was soon demonstrated that a number of patients treated in this manner subsequently recurred in a femoral node and died [6]. The problems with this approach were confirmed in a prospective Gynecologic Oncology Group (GOG) study [7].

The third attempt involved the use of radiation therapy to avoid groin dissection altogether. For patients with no clinically suspicious groin nodes, the GOG conducted a randomised, prospective study of bilateral groin radiation versus inguino-femoral lymphadenectomy (and post-operative radiation for patients with positive groin nodes) [8]. The study was stopped prematurely because there was a recurrence rate of 19.2% (5 of 26) in the radiation arm, versus 0% in the surgical arm. Although the radiation technique in this study has been criticised [9], no other prospective study to address this issue has ever been undertaken.

Finally, the concept of sentinel node biopsy was introduced in 2008 [10]. This procedure is associated with a much lower incidence of lymphedema. Although the methodology for sentinel node dissection has evolved over time, with increasing utilisation of indocyanine green for mapping [11], the procedure of sentinel node biopsy is still associated with groin recurrence in a small but definite number of cases [11,12,13,14,15,16]. Most patients who have a clinical recurrence in an un-dissected groin die of disease, in spite of active management [17]. However, following the groins of patients with a negative sentinel node (s) with serial ultrasonography [18] and detecting positive nodes before they become clinically apparent should make sentinel node biopsy universally accepted as a safe and effective plan of management.

Most clinical management guidelines recommend limiting the sentinel node procedure to patients whose primary cancer is 4 cm or less in diameter. With a view to determining the feasibility of decreasing the risk of lymphedema for a larger group of patients, without compromising survival, we conducted a prospective study in which we separated the inguinal nodes into a medial and a lateral group, based on the lateral border of the femoral artery. The hypothesis was that nodes lateral to the femoral artery would not be involved if nodes medial to the artery were tumour-free.

## 2. Materials and Methods

Patients undergoing either a complete inguino-femoral lymphadenectomy, groin lymph node debulking, or sentinel node biopsy for vulvar cancer were prospectively selected for inclusion in the study. We included patients with unifocal or multifocal tumours of any size or any histological type, including those with extension medially to involve the urethra, anus, or vagina. We initially excluded patients whose tumour extended (i) laterally beyond the labiocrural fold onto the thigh, or (ii) posteriorly beyond the perineum onto the buttocks. We subsequently also excluded patients whose tumour extended (iii) anteriorly onto the mons pubis, after encountering one patient with invasive Paget’s disease of the vulva, whose tumour extended to the lateral mons pubis. This patient had one positive lateral inguinal node, and negative medial inguinal and femoral nodes.

After obtaining informed consent, patients with no palpably enlarged groin nodes underwent a complete unilateral or bilateral inguino-femoral lymphadenectomy. The inguinal nodes were divided into a medial and a lateral group, based on the lateral border of the femoral artery, and sent for histopathology in separate bottles. The femoral nodes were also sent in a separate bottle. If bulky nodes were present, we obtained a pre-operative CT scan of the chest, pelvis, and abdomen. All bulky groin nodes were removed and sent for frozen section. The location of the bulky groin nodes in relation to the lateral border of the femoral artery was noted. If at least one bulky node was positive, complete inguino-femoral lymphadenectomy was not performed. This was based on our previous experience with the management of bulky positive groin nodes [19], which was subsequently confirmed by Nooji et al. [20]. If there were any enlarged pelvic nodes on CT scan, these were resected via an extraperitoneal approach through the groin incision. If the patient had a smaller cancer and gave informed consent for sentinel node biopsy, the relationship of the sentinel nodes to the femoral artery was recorded. If there was any evidence of cancer cells in a lymph node, it was regarded as being positive.

Our technique for inguino-femoral lymphadenectomy has previously been described [17]. In brief, a linear incision is made about 1 cm above and parallel to the groin crease. The subcutaneous tissue is incised down to Camper’s fascia, and the latter is then incised. The inguinal nodes are removed by dissecting the fat from the femoral triangle between Camper’s fascia and the fascia lata, while preserving both fascial layers. The lateral border of the femoral artery is determined by palpation of the artery. The femoral nodes, part of the medial compartment, are removed by removing the fat medial to the femoral vein in the fossa ovalis.

Bilateral groin and pelvic radiation were given to all patients with extracapsular spread, bulky positive nodes or 3 or more micro-metastases (≤5 mm). All patients were followed for a minimum of 33 months, and no patient was lost to follow-up. No patient recurred in the groin.

### Statistical Methods

The association between the positivity/negativity of a medial inguinal node and the likelihood of having a positive lateral inguinal node was tested using Fisher’s exact test. The probability of having a positive lateral node given a negative medial node was estimated from the logit of a logistic regression model of lateral node positivity as the outcome with medial node positivity/negativity as the predictor variable.

The observed number of cases of lateral lymph node metastases accompanying medial lymph node micro-metastases was used to estimate Poisson probabilities of the finding against a nominal range of expected numbers of such cases, since the latter was not well established because of the limited number of cases.

## 3. Results

Between December 2010 and July 2018, 76 patients underwent some form of groin node dissection, and data were obtained from 112 groins. The clinico-pathological characteristics of the patients are shown in Table 1. Fifty-nine patients (77.6%) had complete inguino-femoral lymphadenectomy, of whom twenty-four had a bilateral dissection. Thirteen patients (17.1%) had resection of bulky positive nodes only, of whom eight had a bilateral dissection, one patient (1.3%) had bilateral sentinel node biopsies (which were negative), and three patients (4%) had a unilateral inguino-femoral lymphadenectomy and a contralateral sentinel node biopsy. The latter three patients had large unilateral primary tumours which came within 1 cm of the midline.

The number of nodes removed from medial and lateral inguinal regions, and from the femoral region, for the various operations is shown in Table 2. For patients having a complete inguino-femoral lymphadenectomy, there was a median of 5.3 medial inguinal, 3.2 lateral inguinal, and 1.5 femoral nodes removed.

The status of the groin nodes and the location of positive nodes is shown in Table 3. Positive groin nodes were found in 29 patients (38.2%) and 37 groins (33.0%). All patients with positive nodes had positive nodes medial to the femoral artery, including two patients (2.7%) who had positive femoral nodes. Both patients with a positive femoral node had a nodal debulking procedure and also had a bulky positive medial inguinal node.

**Figure 1:**
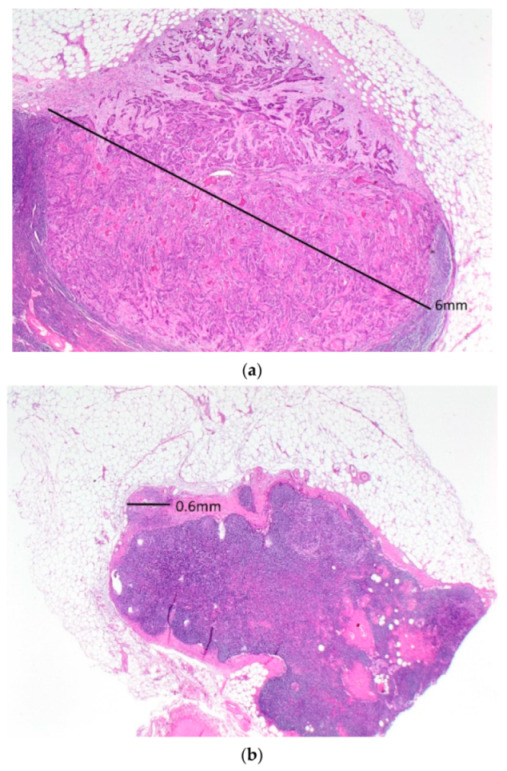
A low-powered view of the positive medial (**a**) and lateral (**b**) inguinal lymph nodes in the only patient with a positive lateral inguinal lymph node who had a complete inguino-femoral lymphadenectomy. Note the much larger metastatic deposit in the medial compared to the lateral inguinal node.

Five patients (5.5%) had positive lateral inguinal nodes (Table 4). All five patients had only one positive lateral inguinal node. In four of the five patients, there were palpably enlarged nodes in the groin, and these patients underwent nodal debulking. Two of these patients had multiple bulky positive medial inguinal nodes. The third patient had a 12 mm metastatic deposit medially and a 6.5 mm deposit laterally, while the fourth patient had a 50 mm metastatic deposit medially and an 18 mm deposit laterally. The fifth patient had no palpably enlarged groin nodes and underwent full inguino-femoral lymphadenectomy. She had one of five medial inguinal nodes positive with a 6 mm metastatic deposit (Figure 1a) and one of four lateral inguinal nodes positive with a 0.6 mm metastatic deposit (Figure 1b).

The characteristics of the primary cancer in patients with positive lateral nodes is shown in Table 5.

The association between medial inguinal node positivity and lateral inguinal node positivity was significant (two-tailed *p*-value = 0.0032). The probability of occurrence of a positive lateral node given a negative medial node was estimated to be 0.00002. However, as there were zero groins with positive lateral nodes and negative medial nodes, this estimate was based on a logistic regression model that failed to converge due to zero events in the categories being modelled. Using instead a nominal value of 0.1 as the number of positive lateral nodes in the presence of negative medial nodes, the probability estimate was 0.00133. If the calculation was restricted to the 59 patients having a bilateral or unilateral inguino-femoral lymphadenectomy only, the probability estimate was 0.00156.

With respect to the likelihood of finding a positive lateral inguinal node in the presence of one medial inguinal micro-metastasis (≤ 5 mm), in this study we had five patients with one medial inguinal micro-metastasis, and all had negative lateral inguinal nodes. However, if the expectation from our sample was that all five should have had a positive lateral node, then based on these data, the Poisson probability of finding zero positive lateral nodes would be 0.0067. If the expectation was that four patients would have positive lateral nodes, the corresponding probability of finding none would be 0.0183, and for three expected cases, the probability would be 0.0498.

## 4. Discussion

Classical books on anatomy, such as Gray’s Anatomy, give a detailed description of the lymph nodes in the groin, but do not indicate which nodes specifically drain the vulva and which nodes drain the leg. A 2007 study by Rob et al. on sentinel lymph node mapping using blue dye and radiocolloid Tc^99^ reported that 83.9% of sentinel nodes were situated in the superficial medial and intermediate inguinal chains, and 16.1% were in the femoral nodes [21]. These data suggest that all lymphatic drainage from the vulva went initially to nodes above or medial to the femoral vein. A subsequent Dutch study using SPECT/CT reported that only 7% of sentinel nodes were located lateral to a line drawn perpendicular to the sapheno-femoral junction [22].

In 2020, investigators used CT lymphangiography on 130 lower limbs from 83 fresh human cadavers (including 75 females) to investigate lymphatic drainage pathways from the lower limb [23]. They reported that 72.8% of the lymphatic drainage flowed to the lateral inguinal lymph nodes.

The present study has demonstrated that the inguinal nodes medial to the lateral border of the femoral artery, together with the femoral nodes, which are medial to the femoral vein, are the first nodes involved in patients with vulvar cancer.

Positive lateral inguinal lymph nodes were detected in 5 of 91 groins (5.5%). In four of these cases, there were bulky nodes apparent pre-operatively, and nodal metastases were confirmed on frozen section. One patient with no clinically suspicious groin nodes had a single positive lateral inguinal node after undergoing full inguino-femoral lymphadenectomy. However, the lateral node had a microscopic deposit 0.6 mm in diameter, and she had a metastatic deposit 6 mm in diameter in a medial inguinal node.

Therefore, in this series of 112 groin dissections of various types, no patient had a positive lateral inguinal node who did not already have a positive medial inguinal or femoral lymph node. The chance of having a positive lateral node in the presence of a negative medial node was estimated to be less than 1 in 10,000. If a nominal count of 0.1 cases of positive lateral nodes in the presence of negative medial nodes was used in the logistic regression model, the estimated probability was still only 0.0013 (a little over 1 in 1000), which would be very clinically meaningful.

Because all patients in this series who had a complete inguino-femoral lymphadenectomy had lateral lymph nodes removed, it was not possible to determine the benefit in terms of lymphedema. We found a median of 6.8 nodes (including femoral nodes) medial to the femoral artery, and 3.2 nodes laterally. If the incidence of lymphedema is proportional to the number of lymph nodes removed, medial inguino-femoral lymphadenectomy should reduce its incidence by about 32%. In our reported experience with groin dissection at the Royal Hospital for Women, patients with five or more resected nodes had an incidence of lymphedema of 35.1%, compared to 17% for patients having four or fewer nodes removed (*p* < 0.01) [24].

An earlier study of 50 patients and 86 groins in patients with penile carcinoma prospectively analysed lymphatic drainage patterns using SPECT-CT and evaluated the implications for the extent of the groin dissection [25]. The groin was divided according to Daseler’s five zones [26]. These were obtained by drawing a vertical and horizontal line over the saphenofemoral junction to create four zones, while the fifth zone was directly overlying this junction. No lymphatic drainage was seen to the inferior two regions, and the authors concluded that the extent of inguinal node dissection could be modified in patients with cN0 nodes in order to reduce morbidity. In our experience, more benefit would be gained by eliminating the removal of all nodes beyond the lateral border of the femoral artery, regardless of whether they were situated superiorly or inferiorly.

Recently, there has been increasing interest in evaluating the diagnostic performance of pre-operative PET/CT scan [27] and ultrasonography [28] to determine the status of the lymph nodes in patients with vulvar cancer. There is also interest in radiomics, which is the high-throughput extraction of large amounts of imaging features which are predictive of tumour biology, response to therapy, and prognosis [29]. However, at this stage, baseline imaging studies lack the sensitivity and specificity to detect micro-metastases, and therefore avoid groin dissection in patients with advanced vulvar cancer [30,31].

If these data can be confirmed, medial inguino-femoral lymphadenectomy should become the standard of care for all patients with invasive vulvar cancer who are not eligible for sentinel node biopsy, regardless of the histologic type, the presence of multifocality, or the size of the primary lesion, as long as the tumour does not extend beyond the labiocrural fold onto the thigh, involve the mons pubis, or extend onto the buttocks. The involvement of the urethra, vagina, or anus should not be a contraindication.

It will take more data to know what the best option is for patients with one medial micro-metastasis (i.e., 5 mm in diameter or less without extracapsular spread). At the present time, we would recommend that these patients should have a lateral inguinal lymphadenectomy or be followed for 12 months with serial ultrasonic examinations of the groin every 2 months. In our series, five patients had one medial inguinal node with a micro-metastasis, and the lateral nodes were negative in all cases. However, one patient had a 6 mm metastatic deposit in a medial node and a 0.6 mm deposit in a lateral node, so for patients with one medial macro-metastasis, we would recommend either ultrasonic surveillance or bilateral groin and pelvic radiation.

The main strengths of this study are that all the data were collected prospectively and that it confirmed the original hypothesis. Its principal finding presents a compelling case for changing practice to lessen the burden of treatment-induced lymphedema for patients with large vulvar cancers. Another strength is that the majority of the surgery was performed by the senior author. Its limitations are that the numbers are not large enough to determine the best approach for patients with one or two medial inguinal micro-metastases. In addition, because both medial and lateral nodes were removed in all patients having an inguino-femoral lymphadenectomy, it is not possible to determine in these patients exactly what benefit medial inguino-femoral lymphadenectomy would have in terms of reducing the incidence of lower limb lymphedema.

## 5. Conclusions

In summary, these data strongly suggest that for vulvar cancers of any size or histologic type that do not extend onto the thigh, buttocks, or mons pubis, the removal of inguinal nodes medial to the lateral border of the femoral artery, along with the femoral nodes, is adequate groin dissection, as long as these nodes are negative. Further research is needed to determine whether these results can be reproduced in other centres, whether or not lower limb lymphedema will be significantly reduced by this operation, and ultimately, whether or not cancer survival will be affected.

## Figures and Tables

**Table 1:** Clinico-pathological characteristics of the patients.

Characteristic	Number
Number of patients	76
Median age (years)	71 years (range 34–96)
FIGO Stage	
IB	44
II	2
IIIA	12
IIIB	1
IIIC	13
IVA1	2
IVB	2
Histological type	
Squamous cell carcinoma	70
Melanoma	3
Bartholin gland (adenoid cystic)	2
Invasive Paget’s disease	1
Complete I-F lymphadenectomy	59 patients (83 groins)
Bilateral	24
Unilateral	35
Unilateral LND + contralateral sentinel node	3 patients (6 groins)
Bilateral sentinel nodes	1 patient (2 groins)
Lymph node debulking	13 patients (21 groins)
Bilateral	8
Unilateral	5

Abbreviations: FIGO, International Federation of Gynecology & Obstetrics; IF, inguino-femoral; LND, lymph node dissection.

**Table 2:** Number of nodes removed at inguino-femoral lymphadenectomy (I-F LND) and lymph node debulking.

Type of Lymphadenectomy	Median	Range
Nodes removed at I-F LND per groin (*n* = 83)		
Total nodes	9.4	2–22
Medial inguinal nodes	5.3	1–13
Lateral inguinal nodes	3.2	0–9
Femoral nodes	1.5	0–6
Nodes removed at nodal debulking per groin (*n* = 17)		
Total nodes	3.3	1–7
Medial inguinal nodes	2.5	1–7
Lateral inguinal nodes	0.8	0–4
Femoral node (only one groin had a bulky femoral node)		

**Table 3:** Nodal status versus nodal location.

Nodal Location	Number	Total Groins Dissected	Percent
Groins with positive medial inguinal nodes	37	112	33.0%
Groins with positive lateral inguinal nodes	5	91	5.5%
Groins with positive sentinel nodes	0	5	0%
Groins with positive femoral nodes	2	74	2.7%
Groins with neg medial, pos lateral nodes	0	91	0%

Abbreviations: neg, negative; pos, positive.

**Table 4:** Details of patients with positive lateral nodes.

Patient	Positive Medial Nodes		Positive Lateral Nodes	
	Number	Largest Diameter	Number	Largest Diameter
1 (debulking)	3/3	23 mm	1/2	15 mm
2 (debulking)	3/3	56 mm	1/1	17 mm
3 (debulking)	1/1	12 mm	1/2	6.5 mm
4 (debulking)	1/3	50 mm	1/1	18 mm
5 (I-F LND)	1/5	6 mm	1/4	0.6 mm

I-F LND, inguino-femoral lymph node dissection.

**Table 5:** Characteristics of the primary cancer in the patients with positive lateral nodes.

Patient	Size and Histology of Primary Cancer
1	5 × 3 cm SCC. Posterior midline lesion extending 4 cm into the vagina
2	8 × 5 cm SCC. Left lateral lesion extending up vagina almost to cervix
3	6 cm SCC of Bartholin’s gland
4	8 × 4 cm SCC. Left lateral and posterior lesion extending 2.5 cm up vagina
5	4 cm SCC of clitoris

SCC, squamous cell carcinoma.

## Data Availability

To protect patient confidentiality, ethics committee guidelines do not permit the study data to be publicly available.
